# Perceived causes and diagnosis of febrile convulsion in selected rural contexts in Cape Coast Metropolis, Ghana

**DOI:** 10.1186/s12887-022-03106-7

**Published:** 2022-01-18

**Authors:** Bernard Afriyie Owusu

**Affiliations:** Master of Philosophy, Department of Population and Health, University of Cape Coast, Cape Coast, Ghana

**Keywords:** Health belief model, Seizures, Paediatrics, Social determinants of health, Maternal and child health(MCH)

## Abstract

**Background:**

Febrile convulsion (FC) is a common seizure disorder among children aged 9 months to 5 years. It is usually benign and self-limiting with favourable prognosis. However, in Ghana, FC is commonly perceived as “*not for hospital*” and widely diagnosed and managed at home based on several beliefs and practices which are limited in related literature.

**Objective:**

This study explored the perceived causes and diagnosis of FC in selected rural communities in the Cape Coast Metropolis, Ghana.

**Methods:**

A descriptive phenomenological study design underpinned the study at five selected communities located not more than 2 Kilometres from the University of Cape Coast Hospital. Purposive and snowball sampling techniques were used to interview 42 participants made up of 27 parents, two grandmothers, seven registered traditional health practitioners, four herbalists, and two faith healers in the communities. The data was analysed using QSR NVivo 12.

**Results:**

Three perceived causes of FC were identified – biological, social/behavioural, and spiritual. Biological causes include genetic abnormalities and other underlying health conditions. The behavioural factors include poor childcare practices and nutrition. Spiritual causes include harm caused by evil spirits. The diagnosis of FC were observed prior, during and after FC attack, and these includes high body temperature, extreme body jerking, and disability outcomes respectively.

**Conclusion:**

The perceived causes of FC are interplay of complex natural, social and spiritual factors that were deep-rooted in local socio-cultural beliefs and FC experiences. Unlike the attack stage, pre-attack diagnosis were usually missed, or misconstrued to mean other health conditions. These findings indicate the need to intensify maternal and child health (MCH) education programmes on FC in the study area through improved primary healthcare.

**Supplementary Information:**

The online version contains supplementary material available at 10.1186/s12887-022-03106-7.

## Introduction

The International League against Epilepsy (ILAE) define febrile convulsion (FC) as seizure events in infancy or childhood (usually between 3 months and 5 years of age) which are associated with temperatures above 38 °C but without evidence of intracranial infection. FC in children below 5 years is a common childhood health condition particularly in deprived areas. It is the commonest seizure disorder among children aged 9 months to 5 years [1] in developing countries, compared to about 5% of all children in developed countries [2]. Although, FC is usually benign and self-limiting with favourable prognosis, it was the 8th leading cause of hospital admissions in Ghana with a total of 12,901 children; several incidences are also not reported [3, 4].

FC events are associated with intrauterine, metabolic abnormalities, vaccination related factors [5] as well as other socio-cultural factors [6, 7]. In Ghana and Nigeria, most mothers from rural communities attribute FC to witchcraft, evil spirits and fever [6–9]. Elsewhere in Turkey, the attribution of FC to supernatural spirits are reported as well [10]. Also critical are family history of epilepsy and complex convulsions which are known FC risk factors [11, 12].

Clinical and home diagnosis of FC include febrile confusion, twitching, increased body temperature, breath-holding attacks and evolving epilepsy syndrome [5, 8, 9, 13, 14]. These are frightening and worrying for caregivers, particularly mothers [8, 9]. Elsewhere, traditional healers attribute FC diagnosis to a spiritual force [15]. Poor FC treatment at home can result in several neurological, cognitive and behavioural impairments [5, 16, 17]. However, available studies in Ghana are health facility based [6, 8, 9], and centred on parents’ reflections, neglecting the home setting including deprivation, influence of older relatives and traditional healthcare practitioners who are key illness decision makers in rural contexts. This study therefore explored the perceived causes and diagnosis of FC from a rural context. Also included are parents’ experiences during episodes of convulsion.

### Contextual and theoretical issues

Context matters in the discussion of health and illness. Usually, the context defines the knowledge system that drives the conceptualisation of persons to define the causation of illness. In most local communities, such as the study areas, emic perspectives drives the perceptions of and related practices of community members. These perceptions are usually handed over from the older to the younger generation. Typically, local knowledge relates to three main dimensions which are the natural, social or behavioural and spiritual dimensions. Thus, the perceptions and beliefs about the causes, and by extension, diagnosis of all forms or types of illnesses are underpinned by these three major dimensions, or their constellations [18]. For instance, some local communities and people have the perceptions that diseases could be contracted from genetic factors [5, 19], poor dietary practices and unacceptable social behaviour [20], and curses or witchcraft attacks [6, 9].

From a theoretical perspective, the Health Belief Model has been applied. The HBM was developed by social psychologists in the United States to explain the common failure of people to participate in programmes aimed at preventing and detecting disease [21, 22]. HBM contains key constructs that predicts why caregivers will take actions to prevent, diagnose or treat their children/ward illnesses based on their beliefs about disease causation. The six (6) HBM constructs are perceived susceptibility, seriousness, benefits and barriers to behaviour, cues to action, and more recently, self-efficacy.

Since the early 1950s, the HBM has been one of the widely used frameworks in health belief research.For instance, HBM has been applied to study FC in Arak City [23–25]. In Turkey and Vietnam, HBM was also applied to study convulsion [10, 26]. Thus, parents/grandmothers who regard their children as *susceptible* to FC, believe that FC is severe and can have potentially serious consequences (*perceived seriousness*), believe that a course of FC action would be beneficial in preventing the consequences of FC such as complex seizures, mental retardations, disability and untimely death (*perceived benefit*), and believe the anticipated benefits of taking action which includes perceived benefit, outweigh barriers of action (*perceived cost*), experiences a *cue to action* such as past experience of FC, childhood fever, or reports of FC in the communities, and have the confidence to make personal decisions (*self-efficacy*) regarding childhood conditions are more likely to take actions based on these beliefs. Thus, this study further explores the internal validity of the HBM constructs across multiple participants involved in FC care.

The major limitation of HBM in relation to this study is that, the HBM constructs view parents or individuals as personal decision makers thereby failing to account for behaviours under social and affective control, of which FC is typical. For instance, the role of parental decisions or intentions, and the influence – approval or disapproval that significant others’ may have on the decision/intention.

## Materials and methods

### Study context

**Fig. 1:**
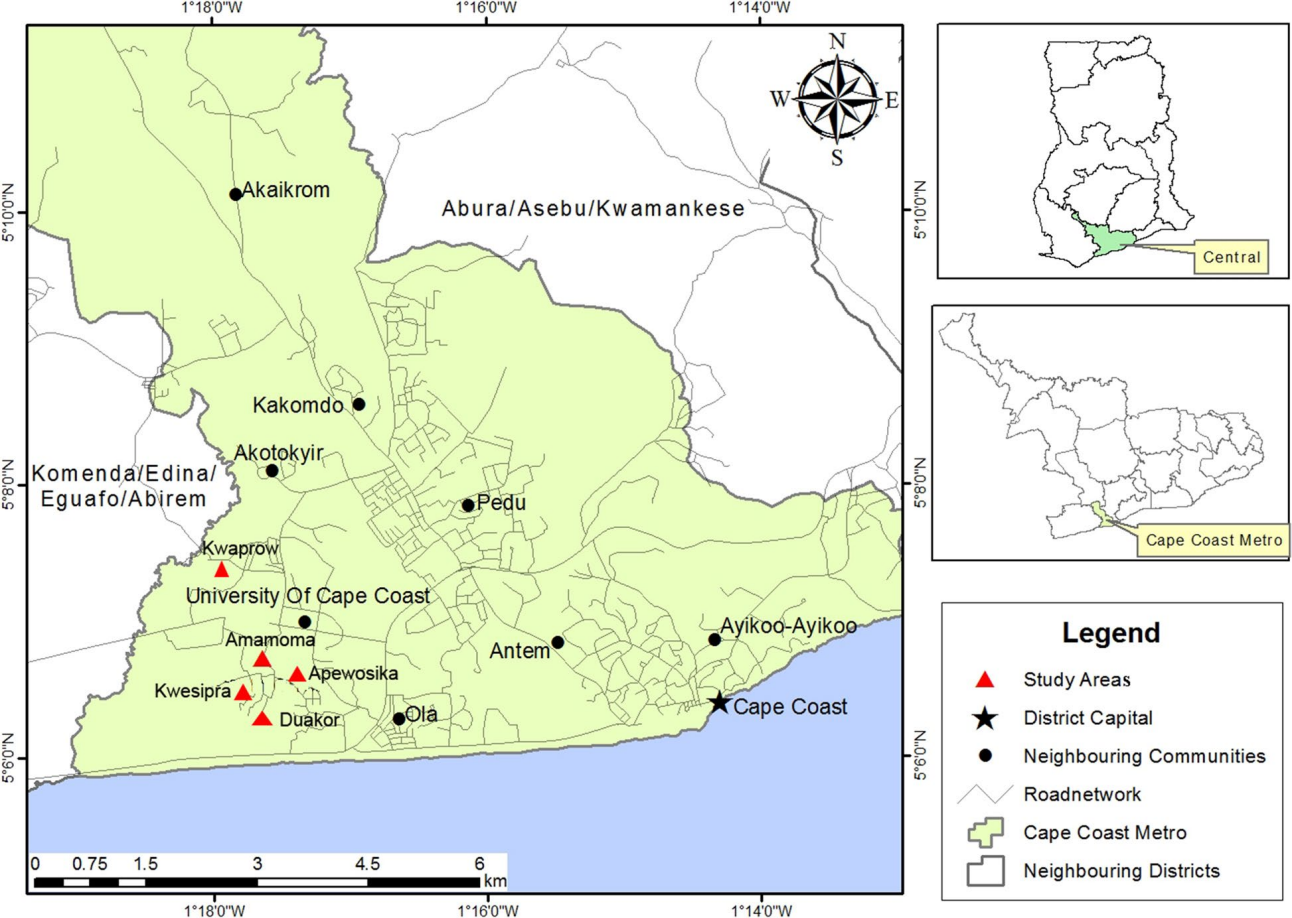
A map showing the 5 selected communities in the study area. Source: Geographic Information System Unit, Department of Geography and Regional Planning, University of Cape Coast (UCC), Ghana. 05th March, 2021

Cape Coast Metropolis is one of the 17 districts in the Central Region, Ghana. The study sampled from five (5) communities which are Amamoma, Kwaprow, Apewosika, Kwesipra and Duakor Communities due to high cases of FC observed in the metropolis. The communities are largely traditional and lack basic social amenities including clean water and sanitation. These communities are predominantly deprived areas, and their major economic activity is fishing. The women are usually fishmongers and traders of farm produce, and the men are into fishing and farming. Few of the communities such as Amamoma and Kwaprow have a poorly resourced Community-based Health Planning Service (CHPS) Center. The communities are within 2 km distance to the University of Cape Coast and Cape Coast Teaching Hospitals. Also resident in these generational communities are traditional health practitioners (believed to have supernatural powers of curing FC through spirits), herbalists, and faith healers who contributes significantly to treating FC. The study area is presented in Fig. 1 below.

### Selection of study participants

Data for the study was drawn from 42 participants made up of 27 mothers, two grandparents, seven traditional health practitioners, four herbalists and two faith healers. Purposive (criterion and maximum variation) and snowball sampling techniques were employed to reach the participants. The traditional healers were the first point of contact who directed field assistants to the homes of their clients. Using a screening form, only caregivers who had treated a convulsing child under age five (5) within the past two (2) years preceding the study were interviewed. The twoyears duration was to reduce recall bias. The study purposively included participants who were directly involved in the treatment of FC to explore their in-depth knowledge, experiences and approaches. To further ensure the collection of reliable data, only traditional health practitioners who were registered under the Ghana Federation of Traditional Medicine (GFTMP) were interviewed. This was because, according to earlier report, traditional medicine still remains in the hands of quack practitioners [27].

### Data collection procedure

Data was collected from 20th November – 13th December, 2020 by two trained postgraduate field assistants. Semi-structured interview guide was developed, pre-tested and used for the data collection. Participants were first identified and interviewed in their homes and treatment centres by making contact with faith healers and traditional health practitioners in the communities. In all, seven participants were exempted from participating in the study as they had not treated convulsion within the past two (2) years, and a participant could not be reached subsequently after scheduling another meeting. The characteristics of these individuals who could not participate in the study however presents no challenge to the robustness of data collected. A maximum of two interviews were conducted in each day. The interviewers had a face-to-face interaction with the participants at their homes and treatment centres. The minimum and maximum duration for the interviews were 45 and 120 minutes respectively. The interviews were conducted in a local language (Fanti or Twi) that were spoken and understood by the participants. Some specific questions on the interview guide include: *What was the cause of your child’s febrile convulsion? Why do you say this was/were the cause(s)? Would you say that every child who suffers from convulsion is as a result of this cause and why?* All the participants consented for the interviews to be tape-recorded. After each day of data collection, all the interviews were transcribed word-by-word into English language, and password protected.

### Rigour

Efforts at ensuring trustworthiness concerned the inclusion of key informants who had treated FC not more than 24 months, interviewing participants at their homes/treatment centres, triangulating data sources and methods, the use of thick descriptions, and confirmation of key evidence by co-practitioners. The reliability criteria also concerned the use of methods that are consistent with related phenomenological studies, using acceptable standards and practices of data analysis and reporting that followed the Consolidated criteria for reporting qualitative research (COREQ) [28]. Independent checking of codes and generation of themes were done and discrepant information were presented as well. To ensure that the findings are credible, the experiences shared resonate with real life experiences of participants, and the methods were triangulated across 5 different participant categories. The study is transferable to local contexts where beliefs, traditional healthcare and socio-cultural norms are deep-rooted. Dependability approaches included independent checking of codes, reporting of discrepant information and the confirmation of the study’s results elsewhere. The methods (data collection and analysis) used in this study is reported to permit the confirmability of the research findings.

### Data analysis and presentation

Data was analysed using QSR NVivo 12. The analysis technique was thematic. My initial approaches followed a five-staged thematic analysis structure [29]. The researcher together with the other two field assistants transcribed the data, organized the data according to the research questions, undertook multiple reading of the transcripts coded the responses and individually generated themes that were later discussed. The use of NVivo 12 for analysis entailed formatting the texts, importation, generation of multiple codes and themes. Key analytical themes that emerged from the transcripts were identified and coded participant responses were categorised under each theme accordingly. The data is presented starting with the socio-demographic characteristics of participants, and followed by focusing on the perceived causes and diagnosis of FC. Specific responses and subjective statements from participants were presented in in-vivo quotations.

### Ethical consideration

Permission for the study to be conducted was obtained from the University of Cape Coast Institutional Review Board [Ethical Clearance ID: UCCIRB/CHLS/2020/42] and the Department of Population and Health, Faculty of Social Sciences, University of Cape Coast, Ghana. The study participants provided verbal consent to willingly participate in the study after the informed consent form was discussed with them. The participants were assured of no financial benefit. However, each traditional health practitioner and herbalist was given a token of Ten Ghana Cedes Only [about $2, USA] for their participation and demonstrations. The token was given after interviews were conducted. All data collection methods were carried out in accordance with relevant guidelines and regulations involved in the conduct of research involving human subjects including the Helsinki Declaration.

## Results

### Background characteristics of study participants

Forty-two participants were interviewed at their homes and treatment centres. In all, 26 mothers, a father, two grandmothers, four herbalists out of which three were women, seven male traditional health practitioners, and two faith healers who were all women participated in the study. Most of the mothers were in their middle ages (30–39 years), and all the herbalists, traditional health practitioners (THPs) and faith healers were over age 50, mostly with no formal education. The THP’s had also practiced traditional medicine for the past four decades. This confirms the finding in Ghana that traditional medicine is in the hands of an illiterate ageing population including quacks [27]. All parents, grandmothers and faith healers professed to be Christians, while herbalists and THP’s were mostly Traditional worshipers. Most of the participants were married with three of the THPs in polygamous marriages. Only six of the participants - all mothers, had attained Senior High School level education. Participants characteristics is summarized on Table 1.

**Table 1:** Background Characteristics of study participants

Characteristics	Participant Categories						
	Mother (26)	Father (1)	Grand-mother (2)	Herbalist (4)	THP (7)	Faith healer (2)	Total (42)
**Sex**							
Female	26	–	2	3	–	2	33
Male	–	1	–	1	7	–	9
**Age Group (in years)**							
20–29	6	–	–	–	–	–	6
30–39	12	–	–	–	–	–	12
40–49	7	–	–	–	–	–	7
50–59	1	1		1	2		5
60–69	–	–	1	2	3	1	7
70–79	–	–	1	1	2	1	5
**Religious Affiliation**							
Christian	26	1	2	1	–	2	32
Traditionalist	–	–	–	3	7	–	10
**Marital Status**							
Married	16	–	–	1	6	–	23
Divorced	9	1	–	2	–	–	12
Widowed	1	–	2	1	1	2	7
**Educational Level**							
No formal educ.	6	1	1	2	5	2	17
Primary	14	–	1	2	2	–	19
SSS/SHS	6	–	–	–	–	–	6
**Ethnic affiliation**							
Akan	24	1	2	3	5	2	37
Ewe	1	–	–	1	2		4
Togolese	1	–	–	–	–	–	1
**Duration of Practice**							
30–39 years	^a^na	na	Na	4	4	–	8
40–49 years	–	–	–	–	3	2	5

Source: Fieldwork, 2020, ^a^na denotes not applicable

### Perceived causes of febrile convulsion

Participants attributed febrile convulsion (FC) to three major causes – natural/biological, social and spiritual. *Natural/biological causes* include high body temperature (mostly malaria), and congenital abnormalities. Most of the parents, particularly the younger mothers who had received junior/secondary level education attributed biological factors such as malaria as the cause of FC. The attribution of high body temperature as a cause of FC was also one of the most common perceptions held by all participants. Some parents expressed how they felt the child’s increased temperature in their palms while baby-sitting. Compared to women with no or primary education, women with junior and senior high school education attributed most of the cases of FC to increased body temperature. The attribution of FC to fever was also confirmed by some faith healers.

#### Congenital causes of febrile convulsion

Congenital factors are pre-existing conditions in the mothers’ womb prior to the child’s birth. Some participants attributed the cause of FC to uterine factors. Only few participants conceived FC to be caused by congenital factors, and these were parents with no formal education and advanced in age. These participants reported that, when convulsions are caused by congenital factors, it is very difficult to treat. However, the study participants could not draw the connection between congenital factors and FC -their expressions indicate some spiritual pathways. For instance, some participants’ including health practitioners’ belief that convulsions are “bought” for a child in her mother’s womb during pregnancy. It was found that genetic perceptions were due to spiritual processes inflicted on the unborn child from external sources rather than through biological/genetic processes.

#### Social/behavioural causes of febrile convulsion

The perceived social causes of FC were grouped into two - poor childcare practices including poor hygiene, and poor childhood nutrition leading to the accumulation of excess phlegm in the lungs. Mothers and grandmothers who were relatively older mostly attributed FC to poor childcare practices. They held the view that poor childcare practices exposes children to unhealthy environmental conditions that triggers FC. Poor childcare practices were found to include mothers’ allowing their children to play on the compound without having an “eye” on them, allowing neighbours to babysit their children, poor sleeping space for children and unhealthy beating of children.

On nutrition, most participants held that FC’s are caused by the accumulation of phlegm as a result of the consumption of unbalanced foods. The perception that phlegm accumulation causes FC was commonly shared among all participants, particularly faith healers, herbalists and traditional health practitioners. The knowledge that phlegm causes FC came about through the treatment or healing process where healers were astonished by the amount of phlegm they had to force out of the child. It was also the commonest theme on the causes of FC in the metropolis.

#### Spiritual causes of febrile convulsion

The attribution of FC to spiritual forces were common amongst grandmothers, traditional health practitioners, herbalists and faith healers, compared to mothers, particularly those who were outside the direct influence of grandmothers, and with no or primary levels of education. These group also represents the older participants. The belief that FC is caused by evil spirits was also general across the traditional healthcare practitioners (THP). An example was when a 54-year-old THP indicated that FC’s caused by evil spirits are *severe and recurrent*. Generally, younger mothers [particularly those below age 35] were not convinced about the extent to which evil spirits could cause convulsion. They usually made references to oral tradition told by their older relatives and neighbours.

The influence of Christian religion on mothers and faith healers beliefs, compared to the beliefs of grandmothers, THPs and herbalists influenced their perception about FC causes. The perception that evil spirits cause FC was overwhelming particularly amongst the older practitioners although some younger participants attributed FC to evil spirits. Traces of social and spiritual spirals particularly from social relations and family structures were found. For instance, participants were of the view that, having a beautiful or handsome child *[defined by culture*] in the community attracted the hate of some relatives/neighbours to harm the child. Harming a child operates through the socio-cultural practices of others back-carrying or carrying children in their arms, and through the foods that children eat. The perceived causes of FC are summarised on Table 2.

**Table 2:** Perceived causes of febrile convulsion

Themes	Perceived Cause of FC	Count
Biological/Natural	High body temperature Congenital abnormalities	31 4
Social	Poor nutrition Poor child care practices	37 6
Spiritual	Evil spirits/Attacks	24

### Diagnosis of febrile convulsion

The main themes that emerged from the diagnosis of FC were pre-attack and during-attack diagnosis. Indeed, during attack diagnosis particularly jerking or twitching was commonly observed amongst participants alike. Prior to the attack, most mothers irrespective of their educational status or category rarely predicted FC. However, compared to mothers without prior FC experience, mothers with prior FC experience mostly predicted FC. Pre-attack conditions such as increased child’s body temperature, no breastfeeding and sleepless nights were noticed by some mothers, yet misinterpreted particularly by mothers with first time experience to mean other health conditions [largely malaria] other than FC. Prior to FC, some mothers observed that their children had mild fever by touching the child’s neck and forehead to feel their temperature. Mothers reported that increase in body temperature were felt from time to time, and were convinced that FCs are caused by increased body temperature.

#### During-attack diagnosis of febrile convulsion

During-attack FC diagnosis was the most apparent. Participants reported a sudden *twitching or jerking, foaming, rolling of eyes, unconsciousness and paleness*. These signs were common across all participants – the commonest being *twitching*. Most mothers also experienced twitching of their children while sleeping *on their laps or in bed*. During FC, mothers observed that their children had *rolling eyes and unconscious* to their environment.

#### During-attack experiences

During FC attack, mothers, particularly those with first time experience went through moments of shock, terror and fright. They approached their first experience by shedding tears and wailing as they rushed outside the home to seekhelp from friends and neighbours nearby. In most instances, their first and second points of call was a neighbours or a passer-by, to an immediate healer – largely, grandmothers, faith healers and herbalist respectively. Similar to first time mothers, participants who had experienced convulsion either as a parent or helped in a treatment process were also terrified. The belief among some participants that only males can hold a convulsing child^1^ also influenced who carried the child for treatment. Friends and relatives who were attracted by the shouts and cries of most mothers were therefore influential in providing immediate care to convulsing children. This was mostly the case for first time mothers in their terror.

#### Post-attack outcomes

After attack and treatment, rare outcome of paralysis and dumbness were also reported. For instance, a father reported that after her daughter’s convulsion, she has become *deaf and dumb*^2^ [The researcher observed this reality]. Also on post-attack outcomes, a 51-year-old mother of eight children, out of which two had experienced FC shared that her child is *paralysed* after having febrile convulsion.

## Discussion

An interplay of complex biological, social/behavioural and spiritual causes of FC were found. Such pluralistic beliefs are predominant in rural-contexts in Ghana. The role of older relatives particularly grandmothers on maternal beliefs and diagnosis of FC cannot be underestimated. Through experiential learning, older relatives are more likely to perceive FC as not severe compared to younger mothers. This implies that the HBM is influenced by participant characteristics including age and social factors. For instance, younger mothers’ beliefs about the causes of FC were heavily influenced by the communities and situations within which they live, particularly with the presence of older relatives and traditional health practitioners who are key cultural bearers and represent important sources of local beliefs. The HBM can therefore be applied to study societal practices by exploring beliefs within a bounded system, and also since traditional healers perform functional roles that satisfies the spiritual needs of parents. The social environment is therefore a major source of beliefs and ill-health diagnosis.

The knowledge that biological or underlying health condition such as fever and congenital abnormalities causes convulsion has been reported in both clinical and indigenous literature. In Arak City, most mothers believed that high fever always leads to seizures [20]. In Ghana and Nigeria, most mothers indicated fever as the main cause of FC [6–8]. The attribution of FC to fever was due to the knowledge that prior to or during FC, most caregivers felt increased child’s body temperature by touching the neck, forehead and feet of the child – a HBM major *cues to action*. Although, fever is a common medical complaint in children, the diagnosis of FC in this contexts transcends fever phobia as it represents parents’ real life experiences and consequently led to convulsion.

An epidemiological study indicated intra-uterine factors, vaccination, and metabolic abnormalities as factors associated with congenital causes of FC [5]. In Bangladesh, neonatal convulsions were caused by maternal complications during pregnancy including septicaemia and meningitis [30]. Congenital factors were also confirmed in a classical study [31]. The aetiology of FC was also found to be dependent on several genetic susceptibility that can be transmitted through parents [32].

The social causes of FC generally depicts childcare practices and nutrition. Febrile convulsion was attributed to social determinants of health specifically poor sanitation, nutrition, and childcare practices. Poor environment during pregnancy for instance is a known determinant of febrile convulsion [20]. On nutrition, particularly phlegm accumulation, most mothers attributed the cause of FC to phlegm in Ghana [6]. Such attribution is due to caregivers’ common experiences with phlegm during episodes of FC treatment.

The belief that FC is caused by evil spirits have been documented in several studies. In Nigeria, 7 out of 10 mothers from rural communities, and 3 out of 10 urban mothers attributed the cause of FC to witchcraft and evil spirits [7]. In Ghana, about 35% of mothers reported that FC is caused by spiritual factors [8], and a related study also showed that 54% of mothers attributed FC to witchcraft and evil spirits [9]. Nyame-Annan also found that mothers attributed the cause of FC to spiritual forces [6]. Compared to faith healers, the traditional healers and older relatives who were traditional worshippers attributed most convulsion cases to evil spirits, and younger mothers were not certain about the extent to which evil spirits caused FC. The attribution of FC to evil spirits have been confirmed in other studies as well [15]. Thus, societal change as a result of education, globalisation and improved access to maternal healthcare services has the potential to shape perceptions and beliefs about disease causation.

Few participants indicated pre-attack diagnosis of FC amidst increased child body temperature and failure to breastfeed. Most mothers therefore could not pre-empt convulsion. For instance, children’s failure to breastfeed was attributed to malaria, rather than convulsion. In Palestine, parents applied antipyretics to prevent or alleviate fever [33]. Other diagnosis include discomfort, food avoidance, and sleeping problems which were all confirmed in this study. Most parents recognised convulsion during the twitching phase, a stage of diagnosis that is widely confirmed in several studies [9, 13, 14].

Mothers, particularly first time mothers went through several mental and emotional trauma as they watched their children go through near-death experiences. In Turkey, parents were shocked observing their child during FC and considered it as life-threatening [23]. These feeling occur due to the fear of FC complications including epilepsy and child disability or mortality [34]. In other reports, the fear of death, seizure re-occurrence, paralysis, mental retardation, physical disability and an uncertain future for the child were some sources of fear for parents [35, 36].

Parents whose children had long-term disability due to convulsion were those whose children had complex FC’s. All such participants also reported to have a family history of convulsion. In other studies, the most consistent FC risk factor is a family history of febrile seizures [11, 12]. Although, the current evidence is not to associate family history of convulsion to complex convulsions and consequently experiencing deformities, complex febrile convulsions can lead to epilepsy [37], as well as several neurological, cognitive and behavioural impairments [5, 16, 17].

### Strength and limitations of the study

The study triangulated data sources across 5 categories of participants who had in-depth knowledge and experiences of FC in the study area. Data was collected through face-to-face interview with 42 participants in their homes and treatment centres. Theoretically, the constructs of the HBM were inherently tested, and the potential to apply such individual based theory to study societies that share similar socio-cultural characteristics is apparent. The findings are however non-generalisable to areas and contexts not considered in this study.

### Implications for policy, practice and future research

The perceived causes and diagnosis of FC in rural contexts were influenced by several socio-cultural beliefs and practices which were handed down from the older to the younger generation. These findings have implications for policy, practice, and future research. Maternal and child healthcare services (MCH) such as maternal education should consider the social-contexts within which caregivers live by identifying potential knowledge adopters and otherwise. Maternal knowledge and beliefs on FC can be shaped during antenatal and postnatal care visits, as well as through mobile clinics in remote areas. There is also the need to intensify PHC services, particularly via the Community-based Health Planning services (CHPs) in deprived areas. Future researches could benefit from exploring the role of older relatives such as grandparents on maternal knowledge and beliefs about disease causation in rural and communal contexts.

## Conclusion

The perceived causes of FC are interplay of complex natural, behavioural and spiritual factors which were deep-rooted in local socio-cultural beliefs and FC experiences. These interplay are such that they influence each other. Younger mothers’ beliefs about the causes and diagnosis of FC are heavily influenced by ingrained social norms and older relatives. Unlike during-attack diagnosis, pre-attack diagnosis of FC are usually missed or misconstrued, and during-attack diagnosis evoked parental shock which influenced the reliance on neighbours. Post-attack outcomes such as difficulties in speaking, hearing or walking  were reported as well. These findings indicate the need to intensify maternal and child health (MCH) education on FC in rural contexts through enhanced primary healthcare programmes.

## Supplementary Information

**Additional file 1.**

## Data Availability

The transcribed data and/or analysed during the current study is available upon request from the Department of Population and Health at pop.health@ucc.edu.gh.

## Footnotes

1 Some participants hold the view that the spirit/soul [locally referred to as *kra*] of a child is given to the child by the father/male, and that complex convulsions will occur if the mother carry a convulsing child. As such, males were allowed to carry the convulsion child. In situations where females carried them, they were handed over to a male person on their way to seek healthcare.

2 Two participants expressed their worry about their daughter and son’s disabilities after convulsion and treatment. Such experience may be discrepant, and represents the experiences of these two participants.

## References

[1] Byeon JH, Kim GH, Eun BL (2018). Prevalence, incidence, and recurrence of febrile seizures in Korean children based on national registry data. J Clin Neurology (Seoul, Korea).

[2] Khair AM, Elmagrabi D. Febrile seizures and febrile seizure syndromes: an updated overview of old and current knowledge. Neurology Res Int 2015 Oct; 2015. 10.1155/2015/849341 PMC4677235 26697219

[3] Ghana Health Service. Annual report: 2016. Accra, Ghana.

[4] Ghana Health Service (2013). Annual Report.

[5] Syndi Seinfeld D, O Pellock JM (2013). Recent research on febrile seizures: a review. J Neurol Neurophysiol.

[6] Nyame-Annan MA. Exploring the Beliefs and Practices of Mothers Concerning the Care of Children with Febrile Seizures at Princess Marie Louise Hospital (Doctoral dissertation, University of Ghana).

[7] Ofovwe GE, Ibadin OM, Ofovwe EC, Okolo AA (2002). Home management of febrile convulsion in an African population: a comparison of urban and rural mothers' knowledge attitude and practice. J Neurol Sci.

[8] Konlan KD, Agordoh PD, Konlan KD, Amoah RM. International Journal of Medical Science and Innovative Research (IJMSIR).

[9] Nyaledzigbor M, Adatara P, Kuug A, Abotsi D (2016). Mothers’ knowledge beliefs and practices regarding febrile convulsions and home management: a study in ho Ghana. J Res Nursing Midwifery.

[10] Kayserili E, Ünalp A, Apa H, Asilsoy S, Hizarcioğlu M, Gülez P, Agin H (2008). Parental knowledge and practices regarding febrile convulsions in Turkish children. Turkish J Med Sci.

[11] Miri Aliabad G, Khajeh A, Fayyazi A, Safdari L (2013). Clinical, epidemiological and laboratory characteristics of patients with febrile convulsion. J Comprehensive Pediatr.

[12] Berg AT, Shinnar S, Hauser WA, Alemany M, Shapiro ED, Salomon ME, Crain EF (1992). A prospective study of recurrent febrile seizures. N Engl J Med.

[13] Sadleir LG, Scheffer IE (2007). Febrile seizures. Bmj..

[14] Paul SP, Blaikley S, Chinthapalli R (2012). Clinical update: febrile convulsion in childhood. Community Practitioner.

[15] Taylor I, Berkovic SF, Kivity S, Scheffer IE (2008). Benign occipital epilepsies of childhood: clinical features and genetics. Brain..

[16] Chin RF (2019). The outcomes of childhood convulsive status epilepticus. Epilepsy Behav.

[17] Chang YC, Guo NW, Huang CC, Wang ST, Tsai JJ (2000). Neurocognitive attention and behavior outcome of school-age children with a history of febrile convulsions: a population study. Epilepsia..

[18] Rüdell K, Bhui K, Priebe S (2009). Concept, development and application of a new mixed method assessment of cultural variations in illness perceptions: Barts explanatory model inventory. J Health Psychol.

[19] Khan MA, Murad MA, Rahman AK, Hossain MM (2006). Management of febrile convulsion: An update. ORION.

[20] Syahida JA, Risan NA, Tarawan VM (2016). Knowledge and attitude on febrile seizure among mothers with under-five children. Althea Medical Journal.

[21] Hochbaum, G.M., 1958. Public participation in medical screening programs: a socio-psychological study (no. 572). US Department of Health, education, and welfare, public health service, Bureau of State Services, division of special health services, tuberculosis program.

[22] Rosenstock IM (1974). Historical origins of the health belief model. Health Educ Monogr.

[23] Sajadi M, Khosravi S (2017). Mothers’ experiences about febrile convulsions in their children: a qualitative study. Int J Community Based Nursing Midwifery.

[24] Sajadi Hazaveh M, Shamsi M (2011). Assesment of mothers' behavior about prevention of febrile seizure in children in Arak city: application of the health belief model. J Jahrom University Med Sci.

[25] Mohsen S, Mahboobeh SH (2013). The effect of education based on health belief model (HBM) in mothers about behavior of prevention from febrile convulsion in children. World J Med Sci.

[26] Tran TK. Fever management in children: Vietnamese parents' and paediatric nurses' knowledge, beliefs and practices.2014. (Doctoral dissertation, Queensland University of Technology).

[27] Addy ME. Traditional medicine. Ugspace. 2017.

[28] Tong A, Sainsbury P, Craig J (2007). Consolidated criteria for reporting qualitative research (COREQ): a 32-item checklist for interviews and focus groups. Int J Qual Health Care.

[29] Lacey A, Luff D. Qualitative research analysis. The NIHR RDS for the East Midlands/Yorkshire & the Humber. 2007.

[30] Haque S, Hossain S, Datta M, Quader MM (2020). Etiology and immediate outcome of neonatal convulsions: a hospital based study. Chattagram Maa-O-Shishu Hospital Medical College Journal.

[31] Levene MI, Trounce JQ (1986). Cause of neonatal convulsions. Towards more precise diagnosis. Arch Dis Child.

[32] Vestergaard M, Basso O, Henriksen TB, Østergaard JR, Olsen J (2002). Risk factors for febrile convulsions. Epidemiology..

[33] Zyoud SH, Al-Jabi SW, Sweileh WM, Nabulsi MM, Tubaila MF, Awang R, Sawalha AF (2013). Beliefs and practices regarding childhood fever among parents: a cross-sectional study from Palestine. BMC Pediatr.

[34] Srinivasa S, Syeda KA, Patel S, Harish S, Bhavya G (2018). Parental knowledge, attitude and practices regarding febrile convulsion. Int J Contemp Pediatr.

[35] Jones T, Jacobsen SJ (2007). Childhood febrile seizures: overview and implications. Int J Med Sci.

[36] Kolahi AA, Tahmooreszadeh S (2009). First febrile convulsions: inquiry about the knowledge, attitudes and concerns of the patients’ mothers. Eur J Pediatr.

[37] Nelson KB, Ellenberg JH (1976). Predictors of epilepsy in children who have experienced febrile seizures. N Engl J Med.

