# Supplementary material for: Perceived causes and diagnosis of febrile convulsion in selected rural contexts in Cape Coast Metropolis, Ghana
Source: BMC Pediatr. 2022 Jan 18;22:45. doi: 10.1186/s12887-022-03106-7 (PMC8764833; doi:10.1186/s12887-022-03106-7)
Supplement: Supplementary file 1 — Additional file 1. [file 12887_2022_3106_MOESM1_ESM.docx]

## Perceived Causes of Febrile Convulsion

On high body temperature as a major cause of FC, a 34 year old mother with junior high school education reported that *“Indeed, my child was feverish and wasn’t feeling well…when I took him to the hospital, the doctor said it was fever.* Also on fever, a 42 year old mother whose two children had experienced convulsion reported that “*my first child had a mild fever, however, the second child’s fever was severe*”. Similarly, a 34 year old mother with no education reported that “*before the convulsion her [the child] temperature was slightly high*”. Some faith healers also reported that *“Convulsions are caused by several factors including malaria and fever”.*

## *Congenital Causes of Febrile Convulsion*

On congenital factors, a 50-year old father with no formal education raising a deaf and dumb daughter whom he believe was caused by FC reported that “*the sickness was in the mother’s womb during pregnancy*”. Also, an 80 year old grandmother and herbalist reported that “*If diseases are in the blood, then the child will have it*”.

A 24-year-old mother reported her prior experience with convulsion when she stated that “*one of my uncle’s son had convulsion. Unlike my son, his [uncle’s son] convulsion was very severe and constantly occurred because witches bought it [FC] for him”.*

The uncertain relationship between congenital factors and FC was affirmed when a 54-year old father reported that “*how would i know whether there is a disease like convulsion or evil spirits from my wife’s background that hurt my child during pregnancy?*”. This father went on to confirm the view of the 24-year-old mother by saying that “*such convulsions [those caused by congenital factors] are very difficult to treat*”.

## *Social/behavioural Causes of Febrile Convulsion*

On poor childcare practices, expressions such as “*leaving your child to crawl/play outside under the heat of the sun*” *[a-45-year old mother]* “*refusal to personally take care of the child by giving the child to other people to carry/babysit*” were common issues shared by mothers and grandmothers when discussing about the causes of FC.

Further, parents and grandmothers generally shared that constantly *beating a baby with the palms at their backs [a-45-year old mother]*, and allowing children to sleep on *dirty mats or polluted sleeping spaces* *[a-76-year old traditional health practitioner]* were major conditions that triggered convulsion.

On nutrition, a 42-year old mother narrated that “*Starchy and tasty foods like toffee, rice, cassava etc. eaten unbalanced, increases the phlegm in the body which causes twitching (wasensen)*”. Similarly, a 70-year-old faith healer with 40 years of treating FC explained that:

*Convulsion comes about through phlegm accumulation in the child. When phlegm get concentrated in the child’s body, a slight anxiety or tremor then triggers twitching. I know people say convulsion is caused by evil spirits – although it happens! The underlying cause is phlegm*”.

Again, the knowledge held by parents, grandmothers and faith healers that phlegm was a common cause of FC was also confirmed by the herbalists. For instance, a 54-year old herbalist with 32 years of treatment experience added that “*phlegm gets accumulated in the chest cavity, interrupting breathing and blood flow hence the abrupt twitching.*

## *Spiritual Causes of Febrile Convulsion*

On the spiritual causes of FC, a 50 year old father with no education reported that “*to be honest, I am convinced that my daughter’s convulsion was spiritual*”.

Also, a 33 year old mother shared that, until her encounter with a doctor at the University of Cape Coast hospital, she had always grown to accept that FC’s are caused by evils spirits. She reiterated that “*I had always been thought by my parents and older relatives that evil spirits cause convulsion…although the doctor was angry at me for bringing my child late to the hospital, he told me ‘3soro yareba’ [FC) was caused by fever and phlegm*”.

A 54 year old father shares a story of a typical case of FC’s caused by evil spirits.

*Just last two weeks, some parents from [mentions the name of the community] brought their young girl to me. They narrated that the child twitched in class and they have taken her to the hospital in the past month, yet the convulsion kept occurring. They didn’t know it was caused by an evil attack. I attended to her and now she is fine and having her education.*

The assertion that FC’s caused by evil spirits are recurrent was also confirmed by a 24-year-old mother when she reported that “*FC’s caused by evil spirits occur periodically, and treatment approaches are difficult”.*

On Christian religion and beliefs about the causes of FC, a 34 year old Christian mother shared that *“no evil spirit can harm my child, I pray to God before and after bedtime and that no evil spirit can bring such evil disease to my child”*.

## Diagnosis of Febrile Convulsion

## Pre-Attack diagnosis

For instance, a 32-year-old mother reported that “*before the attack, she had sleepless nights and cried for about two days. She was not breastfeeding and not taken any food so I thought she had measles.*

Poor pre-attack diagnosis was ascertained when a-32-year old mother reported that “*I did not know that convulsion was about to occur”*.

## During-Attack diagnosis

For instance, a 42-year-old mother reported that “*He was okay when we were going to the farm. The child was carried at my back while harvesting maize in the farm. All of a sudden, I felt a sharp twitching and my child [mentions his name] nearly fell off my back.*

## During-Attack Experiences

For instance, a-39-year old mother reported that “*With the second convulsion, I wasn’t that terrified so I took care of the child at home with the help of my mother*”.

Also, a 28 year mother reported that when she was alerted by her older that and she went inside her room, she saw that her child was “*shaking and his eyes had turned white”.*

## Post-Attack diagnosis

A 50-year-old father expressed his concerns and worries as a single parent when he shared that “*I have to let neighbours take care of her when I’m leaving for work. She’s always alone and can’t speak or hear*, *and I’m worried because nobody understands her like I do”.*

a 51-year-old mother also expressed her worries when she stated that “*Oh the convulsion made him paralyze, and now epilepsy has set in. As you see him smallish like that [points him to the researcher]^[[1]](#footnote-1)^, he’s very old*”.

1. This woman showed her son to me crawling on the floor. I was told the boy had a seizure in school and was brought home. Consequently, he could not receive education beyond lower primary level. Initially, I thought the child was just a boy until she said the boy is 24 years old. The child is paralysed and cannot walk. I see loose muscles with wrinkled hands and saliva all over his body as he crawls towards the kitchen. He looks neurologically impaired [↑](#footnote-ref-1)
